# A potassium chloride to glycine betaine osmoprotectant switch in the extreme halophile *Halorhodospira halophila*

**DOI:** 10.1038/s41598-020-59231-9

**Published:** 2020-02-25

**Authors:** Ratnakar Deole, Wouter D. Hoff

**Affiliations:** 1 Department of Microbiology and Molecular Genetics, Oklahoma State University, Stillwater, Oklahoma, 74078 USA; 2 Department of Biochemistry and Microbiology at Oklahoma State University-Center for Health Sciences, College of Osteopathic Medicine, Tulsa, Oklahoma, 74464 USA

**Keywords:** Biochemistry, Evolution, Microbiology

## Abstract

Halophiles utilize two distinct osmoprotection strategies. The accumulation of organic compatible solutes such as glycine betaine does not perturb the functioning of cytoplasmic components, but represents a large investment of energy and carbon. KCl is an energetically attractive alternative osmoprotectant, but requires genome-wide modifications to establish a highly acidic proteome. Most extreme halophiles are optimized for the use of one of these two strategies. Here we examine the extremely halophilic Proteobacterium *Halorhodospira halophila* and report that medium K^+^ concentration dramatically alters its osmoprotectant use. When grown in hypersaline media containing substantial K^+^ concentrations, *H. halophila* accumulates molar concentrations of KCl. However, at limiting K^+^ concentrations the organism switches to glycine betaine as its major osmoprotectant. In contrast, the closely related organism *Halorhodospira halochloris* is limited to using compatible solutes. *H. halophila* performs both de novo synthesis and uptake of glycine betaine, matching the biosynthesis and transport systems encoded in its genome. The medium K^+^ concentration (~10 mM) at which the KCl to glycine betaine osmoprotectant switch in *H. halophila* occurs is near the K^+^ content of the lake from which it was isolated, supporting an ecological relevance of this osmoprotectant strategy.

## Introduction

All halophilic organisms face the risk of cellular dehydration caused by the high osmotic activity of saline environments, and require osmoprotection strategies to survive. Since saline oceans, saline lakes, inland seas, and saline groundwater constitute ~97% of all water on earth^1^, and salt deposits underlay roughly one quarter of the land on earth^2^, saline and hypersaline environments are of great ecological significance. In addition, salinity has been identified as a major determinant for microbial community composition^3^. Therefore, halophilic adaptations are of general biological interest. Halophilic microorganisms manage to thrive in saline and even hypersaline environments by increasing the osmotic activity of their cytoplasm to match that of the environment. Two types of osmoadaptive strategies are employed by halophiles and extreme halophiles^4–7^: either the accumulation of molar concentrations of KCl or the accumulation of organic compounds such as glycine betaine compatible solutes in their cytoplasm^8–11^. Which of these two main osmoprotection strategies an organism utilizes has profound implications for its ecology, physiology, biochemistry, and evolutionary history.

Organic osmolytes do not interfere with the functional properties of cytoplasmic components^10,12–14^, and are thus referred to as compatible solutes. For organisms utilizing this osmoprotection strategy it therefore suffices to accumulate compatible solutes either by uptake from the extracellular medium or by biosynthesis. However, the biosynthesis of molar concentrations of organic osmoprotectants represents a large investment in terms of ATP, reducing equivalents, and carbon^5^. In contrast, high concentrations of KCl are generally toxic to various cytoplasmic enzymes^15^. In organisms using KCl as their main osmoprotectant the profile of isoelectric points of the entire proteome is shifted to acidic values^16–23^. Such proteome acidity is believed to allow enzyme function to proceed in the saline cytoplasm^19–21,24,25^, but is thought to require the organism to permanently maintain high levels of cytoplasmic KCl. Once an organism has evolved an acidic proteome, a key advantage is that KCl is a bioenergetically inexpensive osmoprotectant. Haloarchaea such as *Halobacterium salinarum* rely almost entirely on KCl, while many halophilic Bacteria predominantly use glycine betaine^26,27^. Almost all halophiles and extreme halophiles for which osmoprotectant levels have been reported exclusively use either KCl or compatible solutes as their main osmoprotectants^4–6^. This overarching view that has guided thinking regarding the dual nature of osmoprotection strategies has been challenged by recent experimental results^28,29^, which prompted us to perform the studies reported here.

Most knowledge regarding halophilic osmoprotection strategies has been obtained through extensive studies of a very small number of organisms, particularly the Archaeon *Halobacterium salinarum*^17^ and *Salinibacter ruber* (Bacteroidetes)^22,30^. Therefore, we aimed to examine the osmoprotection strategy of extreme halophiles from other bacterial phyla, and selected two closely related^31^ extremely halophilic purple photosynthetic Proteobacteria: *Halorhodospira halophila*^32^ and *Halorhodospira halochloris*^33^. While *H. halochloris* has been shown to utilize glycine betaine as its main osmoprotectant^34^, we found that *H. halophila* predominantly accumulates molar concentrations of KCl^28^. This result implies a relatively recent and dramatic evolutionary change in osmoprotection strategy. Unexpectedly, we found that at lower salinity (approximately that of sea water) *H. halophila* retains an acidic proteome but does not accumulate cytoplasmic KCl beyond levels found in *E. coli*^28^. This observation does not follow the expectation that acidic proteomes in halophiles require high levels of KCl to be functional. In addition, further studies revealed that many *Halobacteriales* are not limited to the use of KCl as their osmoprotectant but utilize organic osmoprotectants^29^. Taken together, these observations imply an unexpected diversity in osmoprotection mechanisms. While most extreme halophiles are believed to dedicate their osmoprotection strategy either to the use of KCl or organic osmoprotectants, here we report the existence of an “omoprotection switch” in *H. halophila*, allowing this organism to switch between the KCl and organic osmoprotectant strategies depending on environmental conditions.

## Methods

### Cell growth

*H. halophila* SL1 and *H. halochloris* were obtained from DSMZ, Germany, and were grown in DSMZ 253 medium without yeast extract containing various NaCl (5–35%) and KCl (0.035 to 10 g/l) concentrations. Yeast extract was omitted to avoid possible unknown increases in medium K^+^ concentration and because it contains proline and glycine betaine that can interfere with experiments on osmoadaptation. Anaerobic photosynthetic cell growth was performed in fully filled and closed 20 ml screw cap glass tubes, and monitored through the optical density of the cultures at 660 nm using an HP8453 diode array spectrophotometer using a custom-made adaptor to hold the glass tubes in place in the measurement beam of the spectrophotometer. It should be noted that the use of optical density introduces some uncertainties, because it is affected by the size and shape of bacteria and by the difference in refractive index between the cells and their medium, and these properties can vary during osmoadaptation. Since our experiments involve the determination of growth curves (typically over three days), our data allow us to verify if the cultures exhibit sustained growth (as detected by a sustained increase in optical density).

### Determination of cellular potassium and chloride content

For plasma emission spectrometric determination of cellular K^+^ content, 20 ml of cell culture was centrifuged (3,750 rpm, 25 minutes). Cell pellets were suspended either in isotonic NaCl solutions or in isotonic ammonium sulfate solutions (to avoid Cl^−^ contamination), again pelleted, and dried for 48 hours at 65 °C. The dried pellets were divided in two halves. One half was used to measure potassium and sodium content using inductively coupled plasma emission spectrometry (Spectro Arcos). The second half was used for colorimetric determination of chloride content using the Lachat 8000 Quick Chem flow injection analyzer^35^. The measurements were performed in triplicates in two independent experiments, for a total of 6 measurements per data point.

To calculate cytoplasmic K^+^ and Cl^−^ concentrations for *H. halophila* and *H. halochloris*, measurements on *E. coli* samples were performed in parallel, and were used as a standard to calibrate the measured K^+^ and Cl^−^ amounts in terms of cytoplasmic concentration. This approach uses the published cytoplasmic K^+^ and Cl^−^ concentrations of *E. coli* of 211 mM and 188 mM, respectively^36^. This method uses the assumption that the cytoplasmic volume of *H. halophila* and *H. halochloris* per OD660 unit is comparable to that of *E. coli*. Previously we have reported evidence that the accuracy of this approximation is sufficient to obtain physiologically relevant insights into osmoadaptation by *H. halophila*^28^. It should be noted that errors in value of the cytoplasmic volume of *H. halophila* used here will result in the same systematic error in the absolute concentrations of K+, Cl−, and glycine betaine, and will therefore not affect the main conclusion reported here regarding the existence of a potassium chloride to glycine betaine osmoprotectant switch in this organism.

### Determination of cellular glycine betaine content

Glycine betaine determinations were performed based on published methods^37^ (see^38^ for validation of this method). 100 ml cultures of *H. halochloris* and *H. halophila* cells were harvested in the late exponential phase. Cells were washed using isotonic NaCl solutions and freeze-thawed at −4 °C for 30 minutes to promote cell lysis. The cells were then diluted in distilled water (0.1 mg cells/ml) in the presence of lysozyme (1 mg/ml). 10% (w/v) of 0.2 N perchloric acid was added and the pH adjusted to 7 using a 0.1 N NaOH solution. The resulting cell free extract was passed through the weakly cationic resin Amberlite CG-50 for chromatographic extraction of glycine betaine. The column was eluted with phosphate-citrate buffer pH 5.3. 1.5 ml of 2 N HCl was added to each 0.5 ml fraction collected from this column. 1 ml of reagent (10 g iodine + 12.4 g KI per 100 ml) was then added to convert glycine betaine to its periodide derivative, which has an absorption maximum at 365 nm. The solution was shaken and placed on ice for 20 min. 10 ml of 1, 2 dichloroethane was added followed by thorough mixing. The absorbance of the organic (lower) layer was measured at 365 nm using a Cary 300 spectrophotometer and compared to standard curves of pure glycine betaine between 10 mM to 2000 mM. The cytoplasmic concentration of glycine betaine of *H. halophila* was determined using *H. halochloris* samples grown in 4 M NaCl, which have been reported to contain a cytoplasmic glycine betaine concentration of 1.6 M^34^.

## Results and Discussion

### Potassium limitation prevents the use of KCl as the main osmoprotectant in *H. halophile*

**Figure 1:**
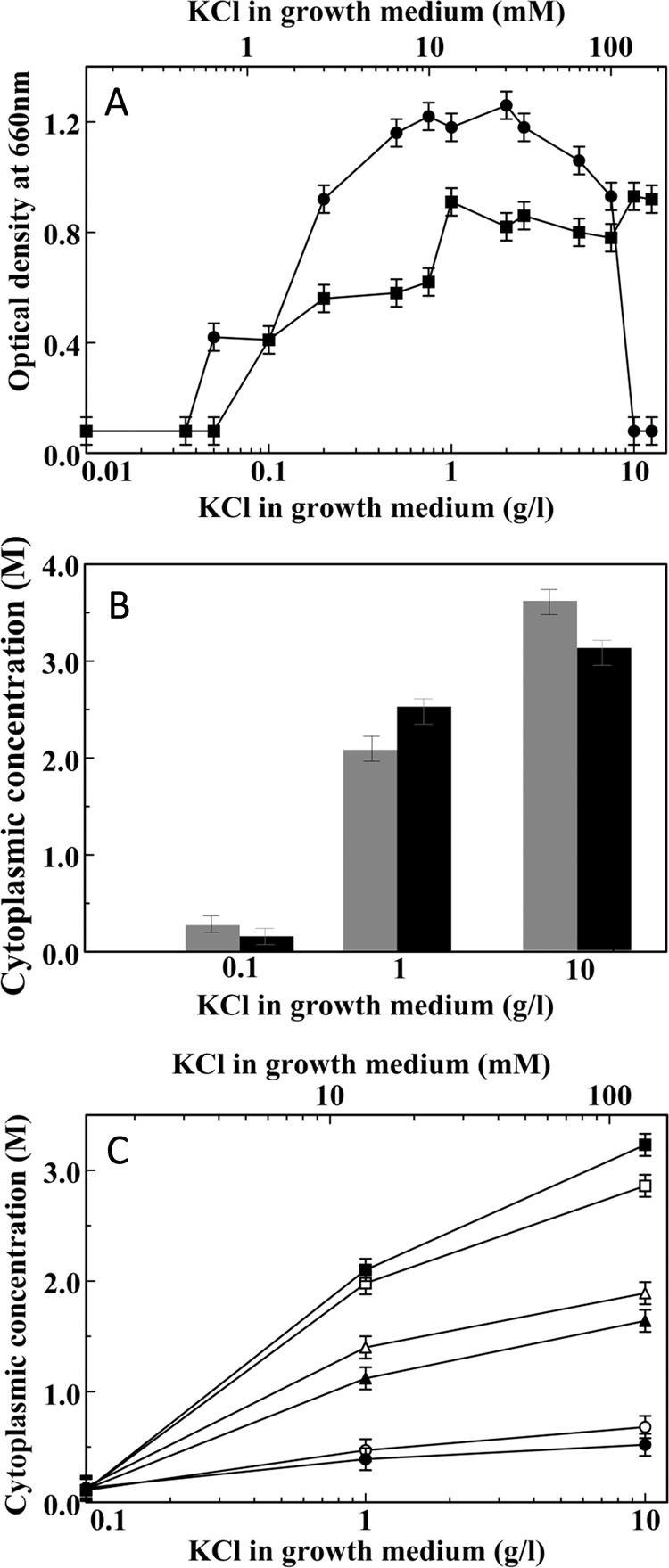
Potassium limitation prevents KCl accumulation by *Halorhodospira halophila*. (**A**) Dependence of the final optical density of *H. halophila* cultures grown at 5% (circles) or 35% (squares) NaCl on KCl concentration in the growth medium. (**B**) Cytoplasmic K^+^ (black bars) and Cl^−^ (gray bars) concentrations in *H. halophila* grown in 35% NaCl in the presence of 0.1, 1, and 10 g/l KCl. (**C**) Effect of growth medium KCl content on cytoplasmic K^+^ (open symbols) and Cl^−^ (closed symbols) concentrations for cells grown in the presence of 35% (squares), 15% (triangles), and 5% (circles) NaCl.

Since *H. halophila* utilizes KCl as a main osmoprotectant^28^, we were interested to examine if reducing K^+^ concentration in the growth medium impedes this strategy. To determine the K^+^ requirement for growth at 5 and 35% NaCl, cell growth was measured in media containing a range of K^+^ concentrations. The expectation for these experiments was that at higher medium salinity *H. halophila* would have a higher requirement for K^+^, and that limiting K^+^ availability would reduce cell growth. Unexpectedly, we observed that for cells grown in media of low salinity, high K^+^ concentrations inhibit growth (Fig. 1A). This cytotoxic effect of high K^+^ concentrations was not observed for cells grown in highly saline media (35% NaCl), and may be caused by osmotic misregulation. Upon lowering medium K^+^ concentrations, cell growth was largely unaffected down to 0.05 g/l KCl (0.7 mM), but was completely inhibited at ~0.02 g/l (0.3 mM) (Fig. 1A). In comparison, the growth of *Halobacterium salinarum* is impaired when the medium K^+^ concentration is reduced to below 3 mM^39^. The K^+^ requirement for growth of *H. halophila* at 35% NaCl is only slightly higher than for growth in 5% NaCl (Fig. 1A).

Based on these experiments we selected conditions to examine the effect of growth medium K^+^ concentration on cytoplasmic KCl accumulation. We osmotically challenged *H. halophila* by growth in media containing 35% NaCl and varied K^+^ concentration from the lowest values that permit substantial cell growth (0.1 g/l KCl), to standard K^+^ concentration (1 g/l KCl) and high K^+^ concentration (10 g/l KCl). Inductively coupled plasma emission spectrometry measurements revealed high cytoplasmic K^+^ concentrations (up to 3.2 M) in cells grown at or above 1 g/l KCl in the growth medium (Fig. 1B). However, when growth medium KCl concentrations were reduced to 0.1 g/l, the cytoplasmic K^+^ concentration dropped sharply to ~0.1 M. Colorimetric assays to determine cellular Cl^−^ levels revealed a very similar pattern for the cytoplasmic Cl^−^ concentration, which ranged from 3.6 M at high medium K^+^ concentration to ~0.1 M at low medium K^+^ concentration (Fig. 1B).

Since medium salinity in our experiments is largely determined by the presence of NaCl, all growth conditions tested offer plentiful Cl^−^. The observation that low medium K^+^ concentrations result in a parallel reduction not only of cytoplasmic K^+^ but also cytoplasmic Cl^−^ concentrations points to the need to maintain overall cytoplasmic electroneutrality, and implies that the main cytoplasmic counterion for Cl^−^ in *H. halophila* is K^+^. Since the growth media contain 35% NaCl (6 M Cl^−^), these cells experience a considerable transmembrane Cl^−^ gradient.

The reduction in cellular KCl content upon lowering the K^+^ concentration of the growth medium was most prominent in cells grown at high NaCl concentrations, where cells were most osmotically challenged. At lower NaCl concentrations a similar trend was observed (Fig. 1C), but maximal cellular KCl levels were lower, in line with the reduced salinity of the growth medium. Taken together, these experiments reveal that growth at K^+^ concentrations below ~10 mM inhibits cellular KCl accumulation by *H. halophila*.

Cells grown in media containing high NaCl concentrations (35%) but low K^+^ levels require a cytoplasmic osmoprotectant to avoid cytoplasmic dehydration. The data depicted in Fig. 1 demonstrate that under these growth conditions cells accumulate only ~0.1 M KCl despite growing in 6 M NaCl. Apparently, an osmoprotectant other than KCl is involved. Interestingly, *H. halochloris* is closely related to *H. halophila*^31^ but utilizes glycine betaine as its main osmoprotectant^34^. Therefore, we examined the glycine betaine content of *H. halophila* cells.

### Regulation of glycine betaine biosynthesis by medium KCl concentration is strikingly different in *H. halophila* and *H. halochloris*

**Figure 2:**
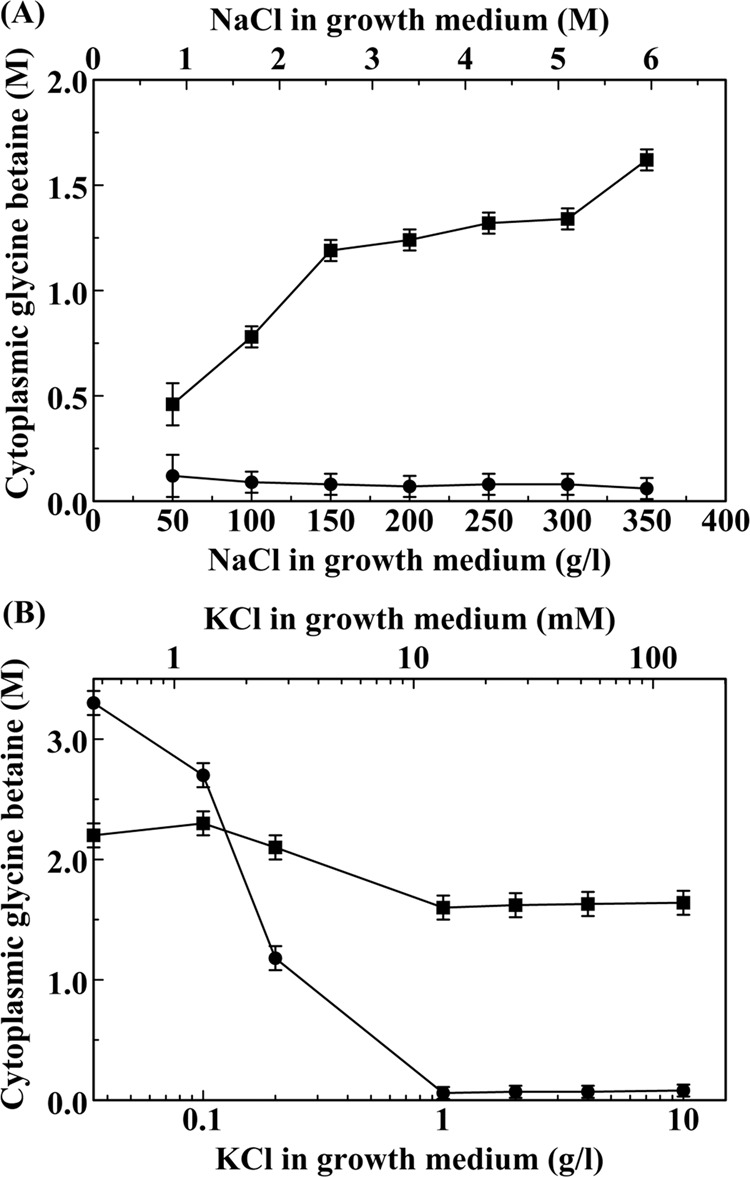
Dependence of glycine betaine biosynthesis in *Halorhodospira halophila* (circles) and *Halorhodospira halochloris* (squares) on growth medium salinity and K^+^ concentration. (**A**) Effect of growth medium NaCl concentration on cytoplasmic glycine betaine concentrations of cells grown in media containing 1 g/l KCl. (**B**) Effect of growth medium KCl concentration on cytoplasmic glycine betaine concentrations of cells grown in media containing 35% NaCl.

We used a colorimetric assay to measure the effect of medium salinity and K^+^ concentration on cytoplasmic glycine betaine concentration in both *H. halochloris*, which accumulates molar concentrations of this organic osmoprotectant^34^, and *H. halophila*, which is known to accumulate molar concentrations of KCl^28^. For *H. halochloris* grown at 1 g/l KCl, these experiments confirmed the presence of high levels of glycine betaine for cells grown in highly saline media (Fig. 2A). Decreasing the medium NaCl concentration from 35% to 5% reduced cytoplasmic glycine betaine concentration from 1.7 M to 0.4 M in this organism (Fig. 2A). In contrast, under these growth conditions *H. halophila* cells contain only ~0.16 M of glycine betaine, even at 35% NaCl. This result reveals a striking difference in the osmoprotection strategy utilized by these closely related organisms. Since the cytoplasmic KCl content of *H. halophila* is greatly reduced upon growth in media containing 0.1 g/l KCl (Fig. 1B,C), we investigated if under these low K^+^ conditions this organism produces glycine betaine.

*H. halophila* and *H. halochloris* cells were grown at 35% NaCl and a range of growth medium K^+^ concentrations. Colorimetric measurements of the resulting cell extracts revealed that the glycine betaine content of *H. halochloris* depends only slightly on medium K^+^ concentration, with an increase from 1.64 M to 2.2 M upon a reduction in medium K^+^ concentration from 10 g/l KCl to 0.035 g/l KCl (Fig. 2B). For *H. halophila* the same decrease in medium K^+^ concentration causes a dramatic increase in cytoplasmic glycine betaine concentration from 0.1 M to 3.3 M (Fig. 2B). The midpoint for this induction of glycine betaine biosynthesis occurs near 10 mM K^+^. These experiments show that *H. halophila* switches its main osmoprotectant from KCl to glycine betaine upon growth in media containing low K^+^ concentrations. For *H. halochloris* such an osmoprotectant switch is not observed; this organism accumulates glycine betaine but not K^+^. These results are in line with the stark difference in proteome acidity between these two organisms: where *H. halophila* exhibits the proteome acidity that is associated with K^+^ accumulation, the proteome of *H. halochloris* does not^28^.

### Genomic potential of *H. halophila* for biosynthesis and uptake of glycine betaine

The availability of the *H. halophila* genome^40^ allows a search for genes likely to be involved in its osmoprotectant strategy. Previously, we reported that its genome encodes multiple K^+^ transporters, in line with its capability to accumulate K^+^ ^28^. In view of the ability of *H. halophila* to utilize glycine betaine as an osmoprotectant reported here, we examined its genome for the presence of genes involved in this capability.

To investigate the genomic potential of *H. halophila* for both the biosynthesis and the uptake of glycine betaine, we performed a series of homology searches for the range of enzymes reported to perform these functions in a range of bacteria. Microorganisms can use variety of routes to increase intracellular glycine betaine concentrations. Glycine betaine can either be biosynthesized or it can be taken up from the external environment. For biosynthesis of glycine betaine either choline or amino acid glycine can be used as a precursor. Choline uptake from exogenous sources is energetically more favorable than its de novo synthesis, and bacteria can use ATP-binding cassette (ABC) transporters or secondary transporters for its uptake. Two choline uptake systems are known in *E*. *coli*: the ABC transporter ProU and the proton motive force-driven, high-affinity uptake system BetT. At low external concentrations, choline is mainly taken up by BetT, whereas at higher concentrations choline is also transported by ProU^41^. In *Bacillus subtilis*, two closely related high-affinity ABC transport systems, OpuB and OpuC, have been identified^42^. OpuC transports a variety of osmoprotectants, whereas OpuB is specific for choline^43^. Soil bacteria, specifically *Arthrobacter globiformis* and *Arthrobacter panescens*, synthesize glycine betaine by one step oxidation of choline by enzyme, choline oxidase with hydrogen peroxide as a byproduct^44,45^. In *Escherichia coli* glycine betaine is synthesized by choline dehydrogenase (CDH) and BADH with the similar betaine aldehyde as an intermediate product^46^. In *Bacillus subtilis* intracellular choline is converted to glycine betaine using two enzymes, glycine betaine aldehyde dehydrogenase (GsbA) and type-III alcohol dehydrogenase (GbsB). These enzymes are encoded by gbsAB operon^47^.

*Actinopolyspora halophila* and *H. halochloris*, two extremely halophilic bacteria, synthesize glycine betaine through a distinct de novo pathway that uses glycine as a precursor, via a three-step methylation process in which S-adenosylmethionine (SAM) serves as the methyl donor. The enzymes involved, glycine sarcosine N-methyltransferase (GSMT) and sarcosine dimethylglycine N-methyltransferase (SDMT), produce the intermediates sarcosine and dimethylglycine and the end product, glycine betaine^48^.

Glycine betaine transporters can be ATP-driven or driven by secondary ion gradients, and belong to three different large families of transporters: (1) ABC transporters (such as ProU from *E. coli* and OpuA from *Lactococcus lactis*), (ii) transporters from the betaine/carnitine/choline transporter (BCCT) family (for example BetP from *Corynebacterium glutamicum*^49^, and (3) major facilitator superfamily (MFS family), see^50^.

**Figure 3:**
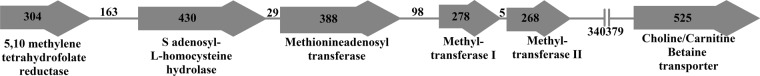
Bioinformatics of glycine betaine biosynthesis and uptake in *Halorhodospira halophila*. The likely operon encoding genes involved in tetrahydrofolate (THF) metabolism and two methyltransferases for the biosynthesis of glycine betaine from glycine are depicted together with the gene encoding a glycine betaine transporter that is located far downstream. The number of encoded amino acids and the number of bases separating the genes are indicated. The sequence similarities of the two depicted methyltransferases and transporter are listed in Table 1.

Homology searches for related proteins encoded in the genome of *H. halophila* yielded the following insight (Table 1, also see Supplemental Table 1). With respect to the biosynthesis of glycine betaine, clear homologs were found in the *H. halophila* genome for the two methyltransferases involved in the pathway starting from glycine (Fig. 3; Table 1). The sequences of the glycine-sarcosine methyltransferases and the sarcosine-dimethylglycine methyltransferases from *Halorhodospira halochloris*^48^ and from the actinomycete *Actinopolyspora halophila*^51^ are highly similar to the corresponding proteins in *H. halophila* (H_hal1677 and H_hal1678) (in the range 72 to 90% sequence identity over the entire length of the protein. These genes appear to be part of the same operon (Fig. 3). Interestingly, immediately upstream of the genes encoding the two methyltransferases are three genes involved in tetrahydrofolate (THF) metabolism. This genetic association suggests that a substantial portion of the methyl groups donated by THF are utilized in *H. halophila* for the biosynthesis of glycine betaine. The presence of this gene cluster in the *H. halophila* genome matches its experimentally observed (Fig. 2) biosynthesis of glycine betaine and provides clear candidates for the genes in the *H. halophila* genome that encode the enzymes for glycine betaine synthesis from glycine. No homologs were found in the *H. halophila* genome for other possible biosynthetic routes for glycine betaine.

**Table 1:** Analysis of the genome of *H. halophila* for the presence of genes involved in the biosynthesis and transport of glycine betaine.

Function	*H. halophila* gene	Organism containing most closely related gene*	Sequence identity
**Glycine betaine biosynthesis**			
Biosynthesis from glycine			
Methyltransferase I	WP_011814464	*H. halochloris*	90%
Methyltransferase II	WP_011814463	*H. halochloris*	73%
Biosynthesis from choline			
Choline dehydrogenase	None	NA	NA
Betaine aldehyde dehydrogenase	None	NA	NA
Choline oxidase	None	NA	NA
**Glycine betaine transporters**			
Transporters from the ABC superfamily			
OpuA	WP_081432241	*Lactococcus lactis*	55%
OpuA	WP_011813050	*Bacillus subtilis*	33%
**Transporters from the BCCT family**			
BetU	WP_011815149	*Escherichia coli*	49%
BetH	WP_011814173	*Halobacillus trueperi*	46%
BetL	WP_011814637	*Listeria monocytogenes*	40%
OpuD	WP_011813054	*Bacillus subtilis*	43%
BetL	WP_011813152	*Listeria monocytogenes*	34%
**Transporters from the MFS superfamily**			
OusA	None	NA	NA
ProP	None	NA	NA

^*^The transporter that showed the highest level of sequence identity to the indicated gene from the *H. halophila* genome is shown.

With respect to the uptake of glycine betaine from the medium through transport systems, the *H. halophila* genome contains seven different genes with clear sequence similarity to relevant transport proteins. Two of these genes are from the ABC superfamily of transporters, and exhibit substantial sequence similarity to OpuA from *Lactobacillus lactis* (55% sequence identity) and OpuA from *B. subtilis* (33% sequence identity), respectively. The remaining five genes showed substantial sequence identify (from 34% to 49%) sequence identity with various glycine betaine transporters from the BCCT family (Table 1). No candidate glycine betaine transporters from the MFS superfamily were identified. Further studies will be needed to test if these seven genes indeed encode transporters for glycine betaine. Taken together, these results suggest that *H. halophila* is capable not only of glycine betaine biosynthesis, but also of its uptake from the growth medium.

**Figure 4:**
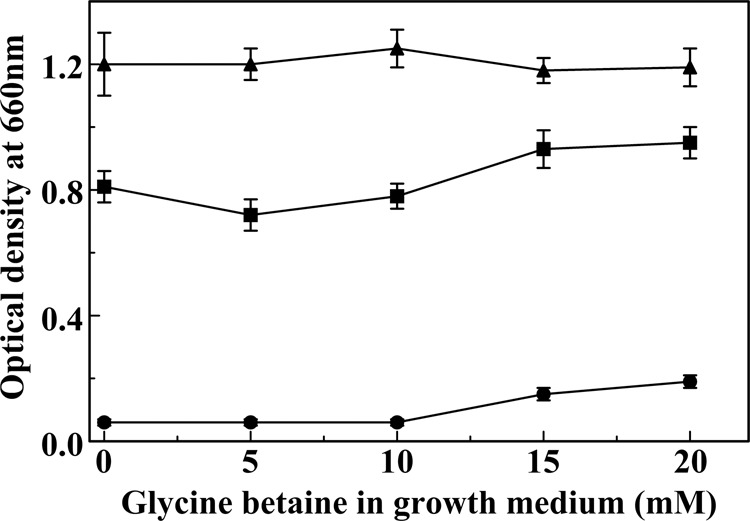
Extracellular glycine betaine stimulates *H. halophila* cell growth at low K^+^ concentrations. The final optical density of cell cultures were determined after 72 hours of growth in the presence of 0.02 (circles), 0.4 (squares), and 1.0 g/l (triangles) KCl in media containing 35% NaCl.

To explore the predicted capacity for glycine betaine uptake by *H. halophila*, we grew the cells at 35% NaCl in the presence of low K^+^ concentrations. These are conditions under which the cells accumulate glycine betaine and therefore would be predicted to benefit from uptake of this compatible solute from the growth medium. Indeed, at reduced K^+^ concentrations that hamper the use of KCl as the main osmoprotectant (0.4 and 0.02 g/l KCl), the addition of 15 or 20 mM glycine betaine to the growth medium results in a modest increase in the final OD of the cultures (Fig. 4). This observation supports the possibility that *H. halophila* can acquire glycine betaine both by de novo biosynthesis and by uptake from the growth medium. However, previous cases in which glycine betaine uptake was an effective part of microbial osmoprotection, glycine betaine concentrations in the growth medium in the micromolar to 1 mM range were found to be effective^14,52^. These considerations indicate that externally provided glycine betaine does not function as an effective osmoprotectant in *H. halophila*.

The natural habitat of *H. halophila* is hypersaline lakes^32,53,54^. It is therefore not immediately obvious how *H. halophila* would encounter environments in which glycine betaine would be available for uptake. Interestingly, when *H. halochloris* and the related organism *Ectothiorhodospira marismortui* are exposed to a sudden drop in medium salinity, as would be encountered in nature after rain causes a sudden influx of fresh water, the cells respond by secreting glycine betaine into the medium^26,55,56^. This glycine betaine can subsequently be taken up by the cells^57^. The presence of a glycine betaine transporter in the genome of *H. halophila* suggests the possibility that a similar mechanism operates in this organism. A related physiological role for glycine betaine uptake is that upon their biosynthesis, a substantial fraction of compatible solute molecules can be released into the growth medium and appears to be recycled via uptake systems, increasing the efficiency of their usage^58^.

### A potassium chloride to glycine betaine osmoprotectant switch

The data reported in Figs. 1C and 2B indicate that growth medium K^+^ concentration has opposite effects on the use of glycine betaine and KCl as osmoprotectants in *H. halophila*. When K^+^ is present at sufficient levels in the growth medium, the cells contain high levels of both K^+^ and Cl^−^, while the biosynthesis and uptake of glycine betaine are reduced to low levels. At low medium K^+^ the opposite effect is observed: cytoplasmic K^+^ and Cl^−^ concentrations are greatly reduced while high levels of glycine betaine biosynthesis are detected (Fig. 2B), together with modest glycine betaine uptake (Fig. 4).

**Figure 5:**
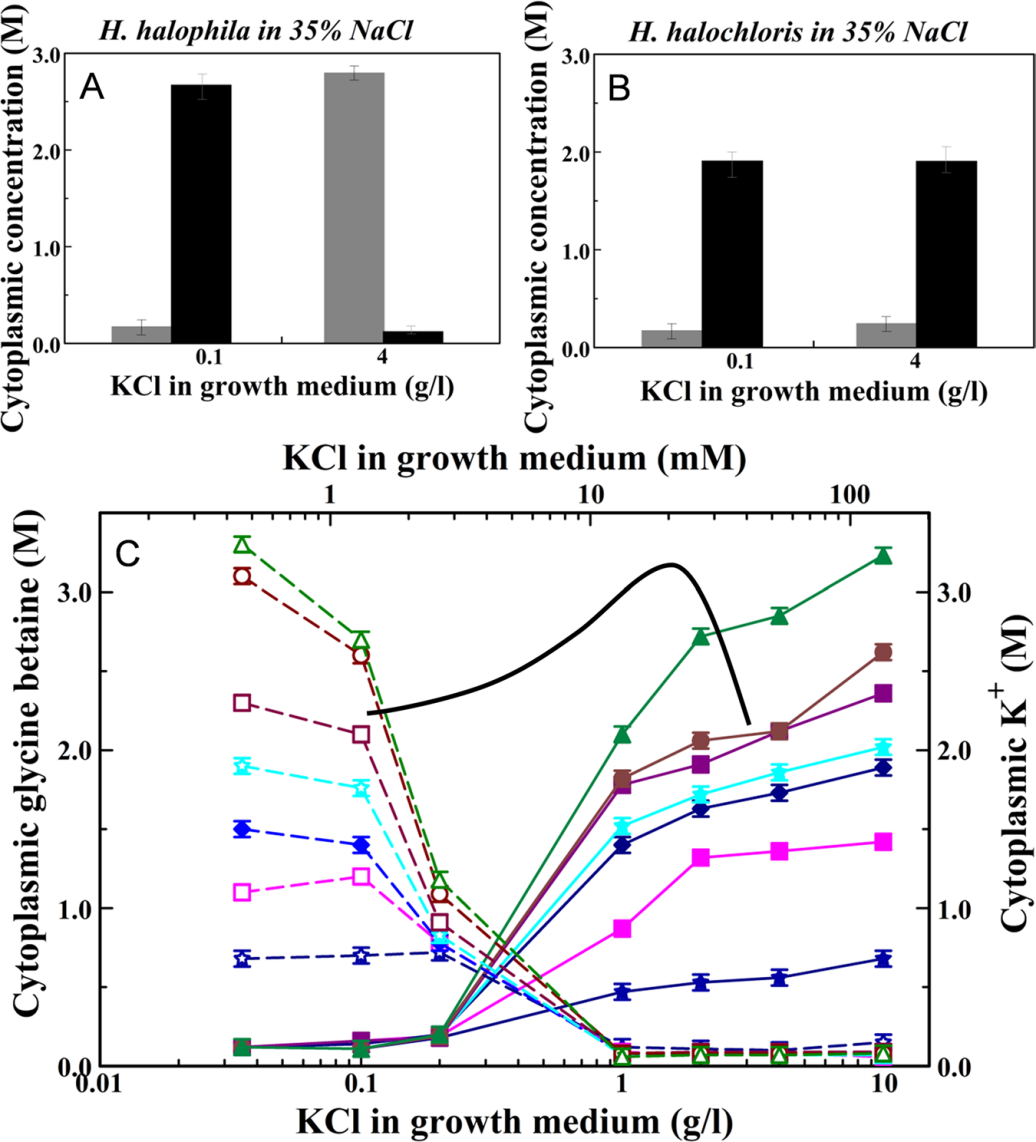
A potassium chloride to glycine betaine osmoprotection switch in *Halorhodospira halophila*. Cytoplasmic K^+^ (gray bars) and glycine betaine (black bars) concentrations of *H. halophila* (**A**) and *H. halochloris* (**B**) grown media containing 35% NaCl and at 0.1 and 4 g/l KCl. (**C**) The effect of growth medium KCl content on cytoplasmic glycine betaine (closed symbols and dotted lines) and K^+^ (open symbols and solid lines) concentrations for *H. halophila* cells growth at various NaCl concentrations: 5% (dark blue diamonds), 10% (pink squares), 15% (dark blue diamonds), 20% (light blue stars), 25% (purple squares), 30% (brown circles), and 35% (green triangles). The average and standard deviation for the KCl content of 6 Wadi Natrun lakes is indicated as a Gaussian curve.

We systematically studied this effect of medium K^+^ concentration on osmoprotectant use at a wide range of medium NaCl concentrations (Fig. 5). These experiments show that below 1 g/l KCl cells use glycine betaine but not K^+^ as a main osmoprotectant, while above 1 g/l KCl the opposite occurs. Thus, near 1 g/l KCl in the growth medium *H. halophila* exhibits a KCl to glycine betaine osmoprotectant switch.

To examine the possible physiological relevance of this osmoprotectant switch, we considered the range of ecologically relevant KCl concentrations that *H. halophila* typically encounters in its natural habitat. For six different Wadi Natrun lakes, in which *H. halophila* thrives^53^, geochemical data have been reported^59^. Interestingly, the average KCl concentration reported for these lakes varies considerably, but on average is close to 1 g/l KCl (Fig. 5). Therefore, depending on the particular Wadi Natrun lake which *H. halophila* finds itself in, it will be exposed to either sufficient K^+^, allowing cellular KCl accumulation, or inadequate levels of K^+^, necessitating the use of glycine betaine as its main osmoprotectant. This analysis implies that the osmoprotectant switch reported here may well be of ecological relevance for *H. halophila*.

Most extreme halophiles for which the osmoprotection strategy has been studied appear to be specialized in the use of either KCl or compatible solutes as their main osmoprotectants^4–6^. Two notable exceptions to this pattern are the extremely halophilic methanogen *Methanohalophilus* strain Z7302, which accumulates molar concentrations of both glycine betaine and K^+^ when grown in hypersaline media^60^, and *Natronococcus occultus*, an organism that combines the use of K^+^ and 2-sulfotrehalose^61^. A novel aspect of the results reported here is that *H. halophila* is capable of switching between KCl and glycine betaine as its main osmoprotectants. Some halophiles exhibit an osmoprotectant switch between different organic osmolotes. Under nitrogen limitation *H. halochloris* reduces its ectoine content and increases its use of trehalose as an osmoprotectant^62^. The moderate halophile *Halobacillus halophilus* switches between different organic osmolytes, including glutamate, glutamine, proline, and ectoine depending on medium salinity and growth phase^63,64^. The osmoprotectant switch reported here for *H. halophila* extends this concept to include the biochemically drastic transition between the two major types of osmoprotectants: KCl and compatible solutes. This increased physiological flexibility of *H. halophila* allows it to utilize the bioenergetically favorable osmoprotectant KCl when conditions allow, while maintaining the capacity to grow in hypersaline media depleted in K^+^.

Observations reported for two other extreme halophiles suggest flexibility in osmoprotectant use depending on medium K^+^ concentrations. In the case of *Halobacterium salinarum* grown at 25 g/l NaCl a reduction in growth medium KCl concentration from 100 mM to 3 mM causes a reduction in cytoplasmic K^+^ concentration from 4.0 M to 2.8 M^39^. Thus, both *H. salinarum* and *H. halophila* respond to K^+^ limitation by reducing their cytoplasmic K^+^ concentration. However, in *H. halophila* this response is much stronger, resulting in a more than 10-fold reduction in cytoplasmic K^+^ content of cells (Fig. 1B,C). For *H. halophila* we found that under these conditions the role of KCl as an osmoprotectant is largely taken over by glycine betaine. In the case of *H. salinarum* it is not known which osmoprotectant compensates for the reduced cytoplasmic K^+^ concent. In this respect it is interesting that *H. salinarum* exhibits chemotaxis responses to various compatible solutes^65^. Secondly, *Salinibacter ruber* was found to contain molar concentrations of KCl in combination with low levels of glycine betaine when grown in media containing high KCl concentrations (5 g/l)^30^. The presence of low levels of glycine betaine in this organism suggests the possibility that upon K^+^ starvation it may increase its reliance on this compatible solute. Thus, the osmoprotectant switch reported here for *H. halophila* may be more widespread, as is supported by recent results indicating that the use of organic osmoprotectants is widespread in the *Halobacteriales*^29^.

## Supplementary information

Supplementary information.
